# Immunoglobulin G4 Smoldering Multiple Myeloma With Immunoglobulin G4‐related Autoimmune Hepatitis: A Rare Case Report

**DOI:** 10.1002/iid3.70244

**Published:** 2025-08-22

**Authors:** Jun Long, Yuchen Shi, Xianyao Wang, Ying Zi, Rongjie Shi

**Affiliations:** ^1^ The Clinical Medical College of Dali University Dali Yunnan China; ^2^ First Affiliated Hospital of Kunming Medical University Kunming Yunnan China; ^3^ Department of Gastroenterology The First Affiliated Hospital of Dali University Dali Yunnan China

**Keywords:** case report, IgG4 smoldering multiple myeloma, IgG4‐related autoimmune hepatitis, IgG4‐related diseases, primary biliary cholangitis

## Abstract

**Background:**

Multiple myeloma (MM) is a common malignant tumor of the hematological system caused by the malignant proliferation of plasma cells, characterized by the production of M proteins and CRAB symptoms. Among them, the Immunoglobulin G MM is the most common, while the IgG4‐MM is extremely rare. Smoldering multiple myeloma refers to a state where there are no clinical symptoms. However, bone marrow plasma cell infiltration reaches 10%–59%, and previously, there were no reports of Immunoglobulin G4 smoldering multiple myeloma (IgG4 SMM) internationally.

**Case Presentation:**

An over‐50‐year‐old woman visited the hospital due to abnormal liver function. Laboratory tests showed a significant increase in serum IgG4 (24.95 g/L), and serum protein electrophoresis detected IgG‐κ M protein (16.05 g/L). A liver biopsy showed IgG4 + plasma cell infiltration (11/HPF) and interface hepatitis. Bone marrow biopsy confirmed IgG4 monoclonal plasma cell proliferation. The diagnosis was IgG4‐κ SMM combined with Immunoglobulin G4‐related autoimmune hepatitis (IgG4‐AIH) and primary biliary cholangitis (PBC). The patient had no typical CRAB symptoms, and no osteolytic destruction was found in imaging. So we formulated a chemotherapy regimen using Bortezomib and dexamethasone, combined with azathioprine for immunomodulation. Unfortunately, after one session of chemotherapy, the patient did not return to the hospital for further evaluation.

**Conclusion:**

This article explores the clinical features and diagnostic challenges of IgG4 SMM coexisting with IgG4‐AIH. IgG4 type SMM needs to be identified with IgG4‐RD. Clinicians should pay attention to IgG subtype detection and clonal plasma cell analysis.

## 1 Introduction

Multiple myeloma (MM) is the second most common malignant tumor in the blood system. In 2018, an estimated 160,000 MM cases and 106,000 deaths worldwide occurred [1]. MM is a malignant disease caused by the malignant proliferation of monoclonal plasma cells, which is related to M protein in serum or urine and organ damage. The main manifestations were hypercalcemia, renal failure, anemia, and osteolytic lesions (CRAB symptoms). Some patients have no clinical symptoms, but if monoclonal plasma cells infiltrate the bone marrow at 10%–59%, it is called smoldering multiple myeloma. Fifty percent of M protein is IgG, 20% is IgA, and < 10% of MM M protein is IgD, IgE, IgM, or double clone [2]. Among the five immunoglobulins, IgG is the most abundant in human serum, which is divided into four subclasses: IgG1, IgG2, IgG3, and IgG4. In the past, some scholars have classified the M protein in MM and monoclonal gammopathy of undetermined significance. As one would expect, IgG1 is by far the most common, and IgG4 is the least [3]. IgG4 MM accounts for about 4% of IgG MM cases [4, 5], and case reports are rare. There have been no reports of IgG4 smoldering multiple myeloma internationally so far.

IgG4‐related disease (IgG4‐RD) is a chronic fibroinflammatory autoimmune disease involving multiple systems and organs. The clinical features are elevated IgG4 in serum and enlargement of affected tissues and organs. The pathological features are IgG4‐positive plasma cells infiltrating tissues and organs, storiform fibrosis, occlusive phlebitis, and sensitivity to steroid therapy [6]. Immunoglobulin G4‐related autoimmune hepatitis can be caused when IgG4‐positive plasma cells infiltrate the liver. IgG4‐AIH is a rare disease.

The simultaneous occurrence of IgG4 SMM and IgG4‐AIH is infrequent and unreported. Here, we report a case of IgG4 SMM with liver damage as the first manifestation, which is rare in clinical practice and has important reference value.

## 2 Case Presentation

A woman over 50 years old submitted an abnormal liver function test to our hospital. She felt a poor appetite, fatigue, and skin itching 1 month before admission. The patient denied fever, bone pain, abdominal pain, etc. There is no family history of liver disease or autoimmune disease, and no long‐term medication history. During the physical examination, the patient was awake, and the orientation ability was good. The conjunctiva was not pale, and the sclera and skin were not stained yellow. There is no tenderness in the sternum. No abnormalities were found in respiratory and cardiovascular system examinations. Her abdomen is soft, with no pain or tenderness. Murphy's sign was negative. Moreover, no neurological impairment was found.

**Figure 1:**
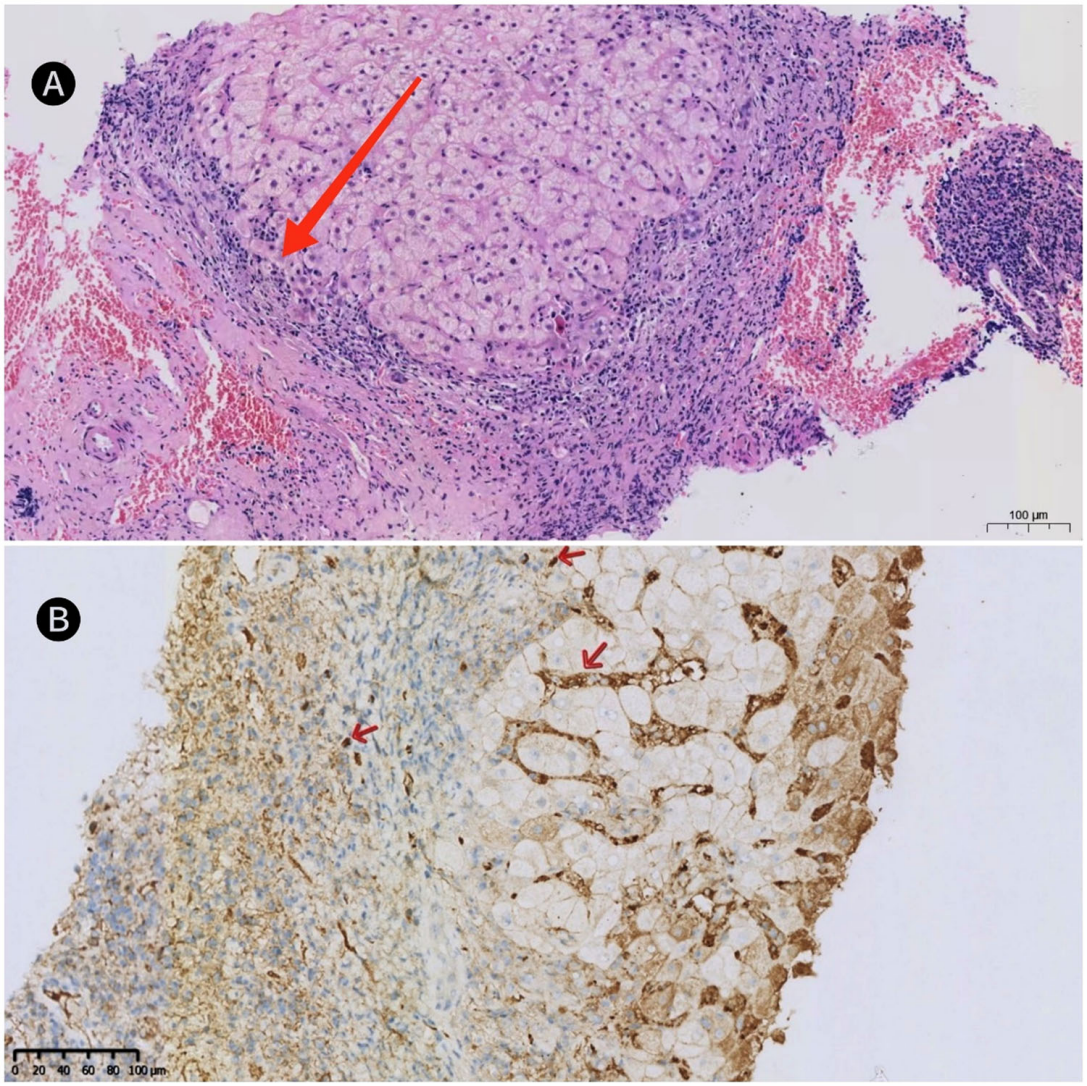
Liver biopsy, (A) the arrow indicates interface hepatitis, H&E staining (× 400). (B) The arrow refers to IgG4+, immunohistochemical staining (×20).

Laboratory examination results at admission (Table 1). Abdominal color Doppler ultrasound: uneven liver echo with splenomegaly. Full abdominal CT scan: the liver surface was uneven, with uneven density in the parenchyma, suggesting cirrhosis; the spleen was enlarged. After a comprehensive evaluation, we performed a biopsy of her liver. Histopathological examination showed plasma cell infiltration and IgG4+plasma cell infiltration (11/HPF). Liver biopsy pathology also found interface hepatitis, rosette‐like liver cells, bile duct hyperplasia, and no amyloid deposition (Figure 1). According to liver biopsy pathology (interface hepatitis, IgG4+plasma cells≥ 10/HPF), serum anti‐mitochondrial‐M2 antibody positive, and serum IgG4 significantly increased, and IgG4‐AIH was diagnosed as overlapping with PBC.

**Table 1:** Laboratory examination results at admission.

Laboratory examination	Concrete value	Reference range
White blood cell	3.01	4–10*10^9^/L
Neutrophil percentage	63.6	50%–75%
Eosinophil percentage	0.3	0.5%–5%
Red blood cell	3.62	3.5–5*10^12^/L
Hemoglobin	123	110–150 g/L
Platelet	140	100–300*10^9^/L
Total bilirubin	42.7	5.1–19 mmol/L
Direct bilirubin	28.9	0–5.1 µmol/L
Alanine aminotransferase	306	0–50 U/L
Aspartate aminotransferase	413	0–50 U/L
Alkaline phosphatase	289	40–150 U/L
Gamma‐glutamyl transpeptidase	256	11–50 U/L
Albumin	25.3	35–55 g/L
Globulin	51.7	20–40 g/L
Total bile acid	133.8	0–10 µmol/L
Urea	5.44	2.86–8.20 mmol/L
Creatinine	46	22–123 mmol/L
β2‐microglobulin	3.80	1.3–2.7 mg/L
Calcium	2.05	2.15–2.55 mmol/L
C‐reactive protein	1.07	0–10 mg/L
Ferritin	193	0–160 µg/L
Erythrocyte sedimentation rate	74	0–20 MM/H
Anti‐nuclear antibody	> 400.00	0–20 RU/mL
Anti‐mitochondrial‐M2 antibody	> 400.00	0–20 RU/mL
Immunoglobulin G	41.8	7–16 g/L
Immunoglobulin G1	17.023	3.824–9.286 g/L
Immunoglobulin G2	4.0192	2.418–7.003 g/L
Immunoglobulin G3	2.5212	0.2182–1.7606 mg/L
Immunoglobulin G4	24.95	≤ 2.0 g/L
Albumin (SPEP)	34.2	55.8%–66.1%
Alpha1 (SPEP)	2.4	2.9%–4.9%
Alpha2 (SPEP)	6	7.1%–11.8%
Beta1 (SPEP)	3.8	4.7%–7.2%
Beta2 (SPEP)	5.1	3.2%–6.5%
Gamma (SPEP)	48.5	11.1%–18.8%
M protein (SPEP)	16.05 g/L	
Kappa light chain	9.8	1.38–3.75 g/L
Lambda light chain	3.55	0.93–2.42 g/L
Kappa/lambda	2.76	1.17–2.93

**Figure 2:**
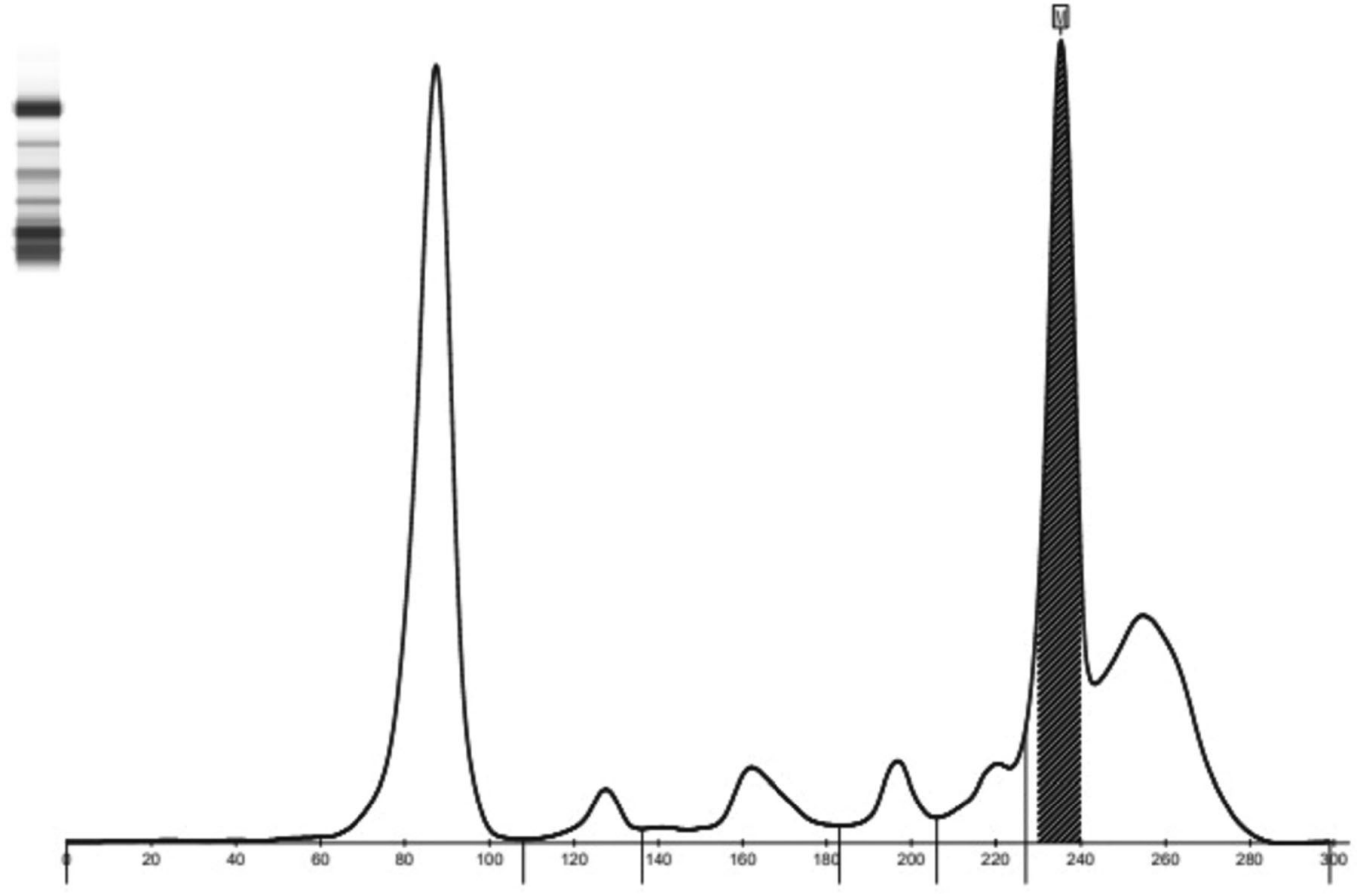
Serum protein electrophoresis, M protein is located in the γ region.

**Figure 3:**
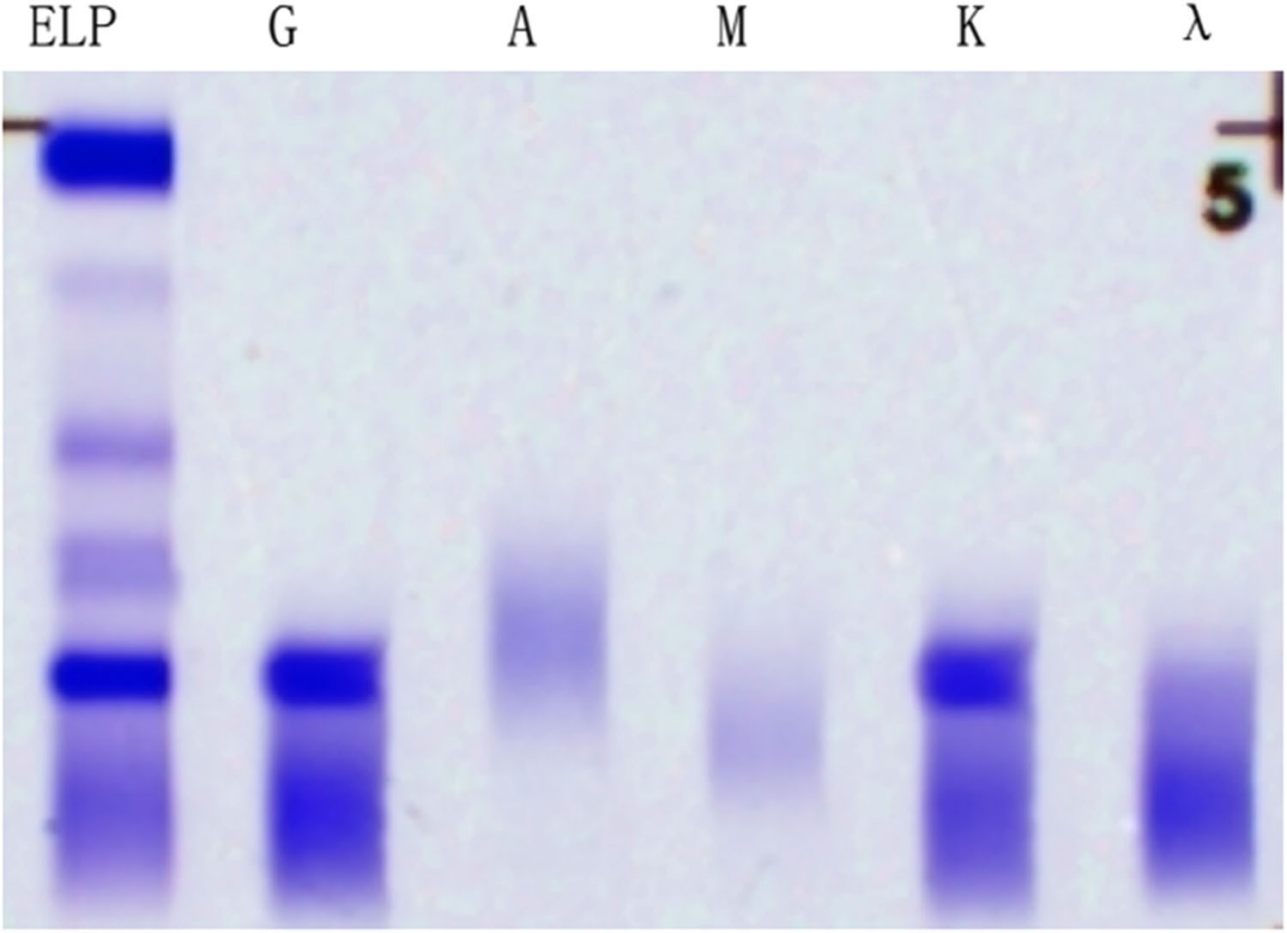
Serum immunofixation electrophoresis indicates a monoclonal immunoglobulin type of IgG‐Kappa.

**Figure 4:**
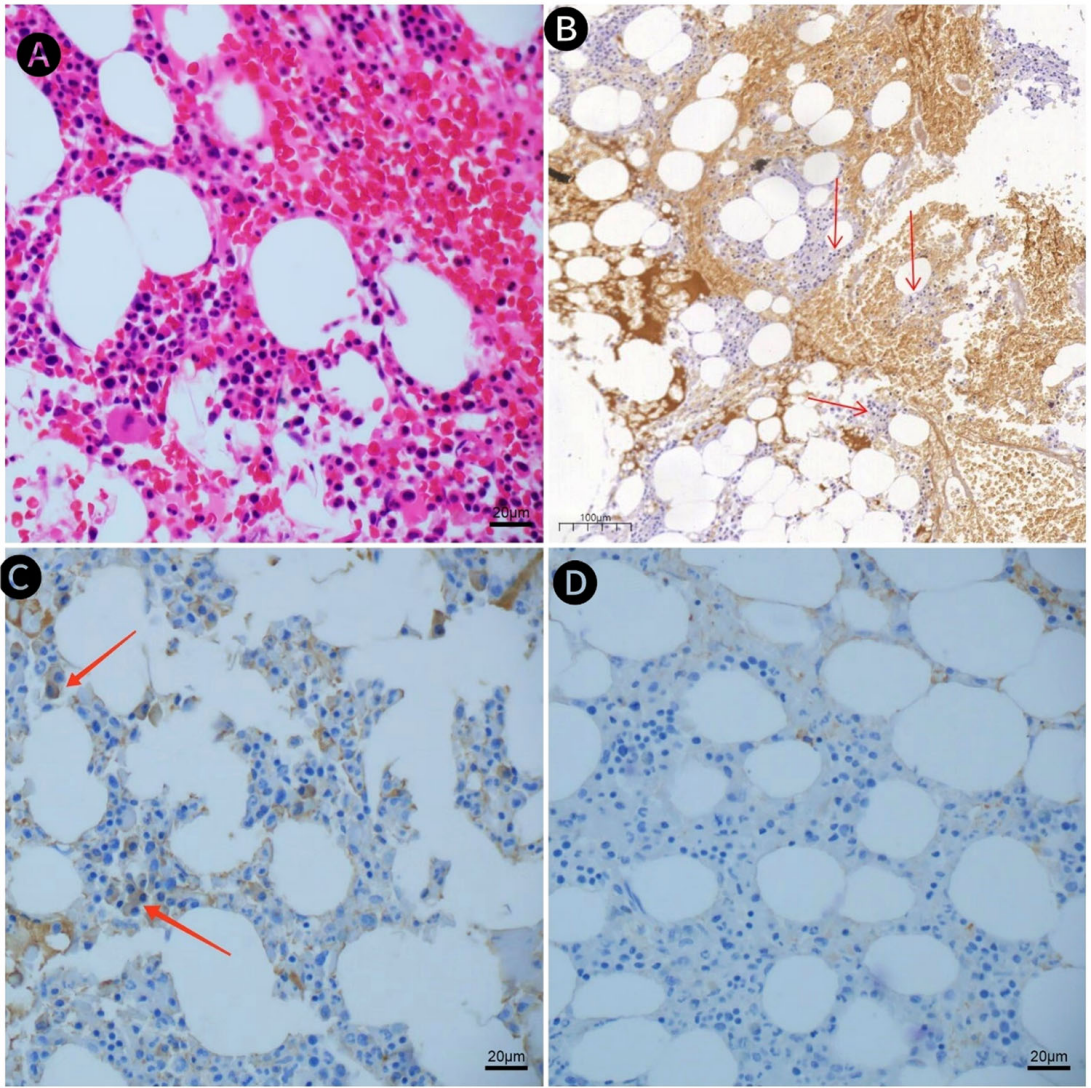
Bone marrow biopsy, (A) H&E staining (×400). (B) The arrow refers to IgG4 +, immunohistochemical staining (×20). (C) The arrow indicates Kappa positivity, immunohistochemical staining (×400). (D) Shows Lambda negativity, immunohistochemical staining (×400).

Serum protein electrophoresis: Albumin 34.2%, Alpha1 2.4%, Alpha2 6.0%, Beta1 3.8%, Beta2 5.1%, Gamma 48.2%, M Protein Percentage 24.1%. Converted to Serum M Protein content 16.05 g/L (Figure 2). Urine protein electrophoresis: Albumin 100.0%, Alpha1 0.0%, Alpha2 0.0%, Beta 0.0%, Gamma 0.0%. Urine protein electrophoresis: M protein was not found. Urine Bence–Jones protein was negative. Serum light chain: Kappa light chain 9.80 mg/L, Lambda light chain 3.55 mg/L, Kappa light chain/Lambda light chain 2.76. Serum immunofixation electrophoresis showed a monoclonal immunoglobulin type of IgG‐Kappa (Figure 3). The concentration of β2‐microglobulin was 3.80 mg/L. The initial perfect bone marrow flow cytometry showed that about 1.22% of monoclonal plasma cells were seen in the specimens. Immunohistochemistry showed plasma cell infiltration (40%), CD138 (+), CD38 (+), CD20 (−), CD3 (−), Kappa (+), Lambda (−), Ki67 (+5%), IgG4+ about 30/mm^2^ (Figure 4). Thus, the patient was diagnosed with IgG4 SMM. Her entire spinal X‐ray, pelvic X‐ray, and skull X‐ray show no signs of osteolytic changes. Head CT scan showed no abnormalities. The non‐contrast CT of the lungs and mediastinum indicated mild interstitial inflammation in both lungs.

Diagnosis: According to all the auxiliary data of the patient, we finally considered the diagnosis of IgG4‐κ type SMM (DS stage I, group A, R‐ISS stage II), IgG4‐AIH, PBC.

### 2.1 Treatment

After obtaining the consent of patients and their families, the treatment plan was formulated. The patient's height was 150 cm, weight was 50 kg, and body surface area was 1.4629 m^2^. Bortezomib 1.9 mg, subcutaneous injection, dexamethasone 20 mg, oral, respectively, on the first, fourth, eighth, and eleventh days. To prevent bone destruction and pathological fractures in patients, we combined pamidronate disodium to prevent bone destruction. We also used a combination of immunomodulator azathioprine, letting the patient take 50 mg daily, and liver anti‐yellowing drug ursodeoxycholic acid capsules, 500 mg orally twice daily. Ask the patient to take the drugs regularly after discharge and regular review. It is a pity that the patient gave up continuing chemotherapy for their reasons after the first chemotherapy. Before the release of anonymous data, the patient's written informed consent was obtained.

## 3 Discussion

The uniqueness of this case is that liver damage (IgG4‐AIH overlaps with PBC) is the first manifestation and lacks typical CRAB symptoms (no bone pain, anemia, hypercalcemia, or renal insufficiency). The patient's serum IgG4 level was significantly increased (24.95 g/L). However, the presence of Bone marrow was infiltrated by monoclonal plasma cells (40%), and IgG‐κ M protein (16.05 g/L) pointed to IgG4 SMM. This case differs from the previously reported IgG4 MM with osteolytic lesions or renal dysfunction as the primary manifestation [4, 5]. In addition, the patient's positive anti‐mitochondrial‐M2 antibody suggested the coexistence of PBC, which further increased the complexity of the diagnosis. Such atypical manifestations may delay diagnosis, highlighting the importance of IgG subtype detection and clonal plasma cell analysis.

IgG4‐related disease (IgG4‐RD) is a chronic, fibroinflammatory autoimmune disease currently believed to be related to the activation of many immune‐mediated lymphocytes. Its clinical features are elevated serum IgG4 and enlargement of affected tissues and organs. The pathological features are IgG4‐positive plasma cells infiltrating tissues and organs, storiform fibrosis, and occlusive phlebitis. It is sensitive to steroid therapy and can affect multiple tissues and organs throughout the body [6]. Identifying IgG4‐type SMM and IgG4‐RD is a clinical difficulty. Although liver biopsy shows IgG4+plasma cell infiltration (11/HPF) and interface hepatitis, IgG4 monoclonal plasma cells in the bone marrow are mainly consistent with diagnosing IgG4‐type SMM. In addition, the patient lacks typical imaging findings of IgG4‐RD (such as pancreatic or salivary gland enlargement), which further supports the diagnosis of SMM. Previous studies have shown that among the 158 patients diagnosed with IgG MM based on bone marrow biopsy, only 6 (4%) were IgG4 MM [4]. Although the authors did not describe the concentration of serum IgG4 in these six patients, none had histological and imaging evidence of IgG4‐RD. In 2020, Japanese scholars found three cases of IgG4 MM patients among 80 IgG MM patients [5], and no evidence of IgG‐RD was found in these three cases. Therefore, it was proposed that monoclonal serum IgG4 was not the primary etiological medium in IgG4‐RD.

IgG4‐related diseases typically present as polyclonal hypergammaglobulinemia, frequently accompanied by β–γ bridging [7]. In 2020, three cases of IgG4 multiple myeloma were documented [8, 9]. The M proteins in these cases were localized to the β region, and no β–γ bridging phenomenon was observed. Additionally, there was no imaging or histopathological evidence of IgG4‐related diseases. The M protein in this case is located in the γ region, and immunofixation electrophoresis indicates it is a monoclonal immunoglobulin of the IgG‐K type. Bone marrow histopathological examination revealed significant Kappa light chain restriction, suggesting the infiltration of monoclonal plasma cells in the bone marrow—a condition uncommon in IgG4‐related diseases. This report details a patient with monoclonal plasma cell infiltration in the bone marrow reaching up to 40%, IgG4 plasma cell infiltration in the bone marrow, presence of M protein in the serum, and absence of CRAB symptoms. Consequently, the patient has been diagnosed with IgG4 SMM.

This case is the first report of the coexistence of IgG4 SMM and IgG4‐AIH/PBC. There may be a potential connection between the two. It is worth noting that although it is still controversial whether IgG4‐AIH belongs to the IgG4‐RD subtype [10], the positive anti‐mitochondrial‐M2 antibody in this case supports the overlap of PBC and IgG4‐AIH, rather than the simple IgG4‐RD involving the liver. Previous studies have reported that there is a specific correlation between autoimmune diseases and cancer, and it may be bidirectional. Autoimmune diseases may increase the risk of certain malignancies [11, 12, 13, 14], possibly due to chronic stimulation of the immune system. Similarly, the incidence of autoimmune diseases in patients with hematological malignancies will also increase [15]. However, the pathogenesis and causal relationship between the two are not clear. It is believed that myeloma cells proliferate in the bone marrow, stimulate osteoblasts to overexpress interleukin‐6 (IL‐6), and then activate osteoclasts, leading to osteoporosis and osteolytic destruction. However, IL‐6 is a multifunctional cytokine that plays an important role in immune response, acute phase response, and hematopoietic regulation. As a pro‐inflammatory factor, IL‐6 may promote an autoimmune response by activating the Th17 pathway and inhibiting regulatory T cells (Treg), thus participating in the co‐morbidity mechanism of IgG4‐AIH and MM [16, 17]. The growth of myeloma cells is regulated by cytokines, among which IL‐6 is the primary growth factor that stimulates myeloma cells and promotes the growth of myeloma cells in MM patients. Secondly, autoimmune diseases are characterized by the body's immune response to its antigens [18]. Therefore, B cells are activated when an autoimmune reaction occurs [19]. Excessive B‐cell activation benefits abnormal B‐cell clones and escapes from standard immune surveillance systems. Multiple myeloma is characterized by uncontrolled proliferation of plasma cells in the bone marrow [20]. It has also been suggested that there may be common genetic susceptibility between autoimmune diseases and plasma cell diseases [21]. Therefore, the above characteristics indicate that there may be a correlation between autoimmune diseases and the development of MM.

## 4 Conclusions

We reported a case of IgG4 SMM with liver damage as the first manifestation, and the patient had no apparent symptoms of CRAB. When the serum IgG4 is significantly increased, it is necessary to actively look for the possibility of IgG4‐RD and malignant tumors, which has important reference value for clinicians. This case suggests that IgG4‐RD and IgG4 SMM should be simultaneously screened for patients with significantly elevated serum IgG4, especially in the absence of typical CRAB symptoms. Detection of clonal plasma cells in the bone marrow and analysis of IgG subtypes are the key to diagnosis.

## Author Contributions

**Jun Long:** investigation, writing – original draft, supervision. **Yuchen Shi:** investigation, writing – original draft. **Xianyao Wang:** data curation, investigation. **Ying Zi:** supervision, resources. **Rongjie Shi:** conceptualization, writing – review and editing, project administration.

## Ethics Statement

The Research Ethics Committee of the First Affiliated Hospital of Dali University obtained ethics approval. Ethics approval number: DFY20241201002.

## Consent

Patients in this study were informed of the research content and signed informed consent.

## Conflicts of Interest

The authors declare no conflicts of interest.

## Data Availability

All data generated or analyzed during this study are included in this published article.
